# Preparation and assessment of an optimized multichannel acellular nerve allograft for peripheral nerve regeneration

**DOI:** 10.1002/btm2.10435

**Published:** 2022-11-01

**Authors:** Tianhao Yu, Qiang Ao, Tianrang Ao, Muhammad Arslan Ahmad, Aijun Wang, Yingxi Xu, Zhongti Zhang, Qing Zhou

**Affiliations:** ^1^ The VIP Department, School and Hospital of Stomatology China Medical University, Liaoning Provincial Key Laboratory of Oral Diseases Shenyang China; ^2^ Department of Developmental Cell Biology, Key Laboratory of Medical Cell Biology, Ministry of Education China Medical University Shenyang China; ^3^ NMPA Key Laboratory for Quality Research and Control of Tissue Regenerative Biomaterial, Institute of Regulatory Science for Medical Device, National Engineering Research Center for Biomaterials Sichuan University Chengdu Sichuan China; ^4^ Cancer Hospital, Chinese Academy of Medical Sciences and Peking Union Medical College Beijing China; ^5^ College of Life Sciences and Oceanography Shenzhen University Shenzhen China; ^6^ Department of Neurological Surgery University of California Davis Sacramento California USA; ^7^ Department of Clinical Nutrition Shengjing Hospital of China Medical University Shenyang China; ^8^ Department of Oral and Maxillofacial Surgery, School and Hospital of Stomatology China Medical University, Liaoning Provincial Key Laboratory of Oral Diseases Shenyang China

**Keywords:** acellular nerve allograft, decellularization, extracellular matrix, multichannel, nerve regeneration, peripheral nerve injury

## Abstract

Peripheral nerve regeneration after injury is still a clinical problem. The application of autologous nerve grafting, the gold standard treatment, is greatly restricted. Acellular nerve allografts (ANAs) are considered promising alternatives, but they are difficult to achieve satisfactory therapeutic outcomes, which may be attributed to their compact inherent ultrastructure and substantial loss of extracellular matrix (ECM) components. Regarding these deficiencies, this study developed an optimized multichannel ANA by a modified decellularization method. These innovative ANAs were demonstrated to retain more ECM bioactive molecules and regenerative factors, with effective elimination of cellular antigens. The presence of microchannels with larger pore size allowed ANAs to gain higher porosity and better swelling performance, which improves their internal ultrastructure. Their mechanical properties were more similar to those of native nerves. Moreover, the optimized ANAs exhibited good biocompatibility and possessed significant advantages in supporting the proliferation and migration of Schwann cells in vitro. The in vivo results further confirmed their superior capacity to promote axon regrowth and myelination as well as restore innervation of target muscles, leading to better functional recovery than the conventional ANAs. Overall, this study demonstrates that the optimized multichannel ANAs have great potential for clinical application and offer new insight into the further improvement of ANAs.

## 1 INTRODUCTION

Peripheral nerve injury (PNI) is the primary type of traumatic damage in the nervous system, affecting over one million people worldwide annually with a high prevalence among younger individuals.^1^, ^2^ Currently, autologous nerve grafting is still considered the “gold standard” for peripheral nerve repair.^3^ However, the clinical application of autologous nerves is greatly restricted by the inherent drawbacks, such as limited availability, morbidity at donor site, and size mismatch.^4^, ^5^ In recent years, numerous tissue engineering nerve grafts made by various synthetic and natural biomaterials have been devised.^6^, ^7^ Because of the insufficient neurorestorative capacity of synthetic biomaterials, they are gradually replaced by natural biomaterials.^8^, ^9^ However, natural biomaterials often contain only one or a few purified extracellular matrix (ECM) components, thus they cannot completely reproduce the complex constitution of ECM related to multiple functions during regeneration.^10^ This makes it difficult for artificial nerve grafts to establish an ideal repair microenvironment. Therefore, acellular nerve allografts (ANAs) have long gained a lot of attention and were regarded as one of the most promising alternatives to autologous nerves.^11^ During the decellularization process, the cellular components capable of inducing deleterious immune responses are removed, but the heterogeneous compositions and inherent ultrastructure of ECM are preserved, which can provide a favorable substrate for nerve regeneration.^12^ Several studies have reported that ANAs have consistently performed better than other types of artificial grafts.^13^, ^14^ So far, there have been commercial ANAs approved by China National Medical Products Administration and U.S. Food and Drug Administration.^15^, ^16^

A variety of physical, chemical, and biological approaches, as a single or combined treatment, have already been developed to prepare ANAs.^17^ Among them, the detergent‐based methods devised by Sondell et al. and Hudson et al. have been extensively recognized and studied. The Sondell method includes repeated treatments with Triton X‐100 followed by sodium deoxycholate (SDC). This method was confirmed to clear cells and myelin sheaths effectively, and the yielded allografts achieved good nerve regeneration after transplantation.^18^ Although nonionic detergent Triton X‐100 is described to cause minimal damage to the native tissues,^19^ SDC, an ionic detergent, is considered to be one of the most potent and disruptive chemical agents. Subsequently, Hudson et al. proposed a milder chemical treatment consisting of several steps repeated twice as well.^20^ The Hudson method has been shown not only to reliably remove cellular components, but more importantly, to achieve superior results in maintaining the ECM intact. This may be attributed to the introduction of the zwitterionic detergents sulfobetaine −10 and −16 in combination with the ionic detergent Triton X‐200. The zwitterionic detergents have properties of both ionic and nonionic detergents, and cause mild damage to ECM during decellularization.^21^ Unfortunately, Triton X‐200, the key detergent of the Hudson method, has been discontinued from the only manufacturer, making it unavailable for further investigation.^22^ Nevertheless, it is worth mentioning that this foundation leads to a wide range of work optimizing the decellularization of nerve grafts.

Currently, despite the Sondell method being proposed early, it is still a well‐accepted conventional decellularization protocol and has been extensively used.^23^ Nevertheless, it has to be mentioned that repeated rinsing in SDC for a long time contributes to the elimination of cellular antigens, but it will inevitably result in substantial loss of ECM components and biomolecules, such as collagen, laminin, fibronectin, and growth factors, which may significantly impair the neuroregenerative function of ANAs.^24^ In addition, it is worth noting that the three‐dimensional organization within peripheral nerves is very compact with low porosity, and mainly consists of small basal lamina tubes.^21^ However, the Sondell method is incapable of remodeling the inherent ultrastructure of native nerves. And the basal lamina tubes remaining within the conventional ANAs do not change remarkably after decellularization.^18^, ^20^ It was reported that the pore size of basal lamina tubes (5–10 μm) is similar to or even smaller than the typical size of regenerative cells (10–15 μm). The basal lamina tubes appear to be not large enough to meet the needs of abundant cell migration and axon extension, which may compromise nerve repair.^25^ More than that, inadequate porosity of ANAs may restrain the diffusion of tissue fluids after transplantation. For this reason, Schwann cells generally fail to completely infiltrate into ANAs, resulting in markedly inferior outcomes.^26^ At present, the conventional ANAs are believed to be incompetent in supporting nerve regeneration across long segmental defects (>3 cm).^27^ Considering these drawbacks in the conventional ANAs, targeted optimization of nerve decellularization methods is a highly prospective research direction, which may open up further opportunities to enhance the pro‐regenerative capacity of ANAs.

In this study, an innovative ANA with optimized axial multiple channels was prepared to deal with the existing problems of the conventional ANAs. A modified decellularization method was developed to obtain the multichannel allografts, which includes a combination of chemical, physical, and enzymatic treatments. To minimize the disruption of bioactive components during decellularization, this newly devised protocol starts with an osmotic shock generated by hypotonic and hypertonic solution, followed by alternating treatments of low concentration Triton X‐100 and 3‐[(3‐cholamidopropyl) dimethylammonio]‐1‐propanesulfonate (CHAPS). CHAPS, as a zwitterionic detergent, can remove cellular components efficiently with minor disruptive effects upon ECM similar to Triton X‐100.^28^, ^29^ The aim of these gentle treatments is to better coordinate the balance of cellular antigen removal and ECM component preservation. Furthermore, to address the shortcomings of inadequate basal lamina tube size and low porosity of the conventional ANAs, unidirectional freeze‐drying was introduced in the processing procedure. In our previous study, unidirectional freeze‐drying has been shown to impart large axially aligned microchannels in ANAs, thereby improving their dense internal ultrastructure.^30^, ^31^ Notably, the freeze‐drying technique itself can be used as a physical decellularization method, effectively lysing cells within tissues and organs.^32^, ^33^ Last, nuclease treatments were carried out to ensure adequate clearance of residual antigenic components within ANAs, allowing them to meet the criteria for in vivo transplantation. The novel ANAs prepared by the modified decellularization method are expected to yield an optimized multichannel architecture as well as achieve superior preservation of ECM components and regenerative factors, resulting in a significant enhancement in neurorestorative effects. As far as we know, there are only a few similar reports on multi‐aspect optimization of ANAs to date. In the present study, the multichannel ANAs were thoroughly characterized on ultrastructural, biochemical, and biomechanical properties in comparison to the conventional ANAs prepared by the Sondell method. Furthermore, their biocompatibility and functionalities on peripheral nerve regeneration were investigated via an in vitro system and a rat sciatic nerve defect model.

## 2 MATERIALS AND METHODS

### 2.1 Animals and ethics

All animal work in this study was approved by the Institutional Animal Care and Use Committee (IACUC) of China Medical University (CMU2019192) and was performed in accordance with Guides for the Care and Use of Laboratory Animals from the Chinese Ministry of Public Health and U.S. National Institutes of Health. Adult male Sprague Dawley rats (250 ± 20 g) were obtained from Changsheng Bio Inc (Liaoning, China). Rats had access to 12‐h light/dark cycles and standard water and food. They were transported and kept more than 7 days before surgery. All surgical procedures were performed under general anesthesia with intraperitoneal injection of sodium pentobarbital (40 mg/kg body weight).

### 2.2 Nerve harvest and decellularization

Sciatic nerves (about 30 mm in length) were harvested aseptically. After the removal of adipose and connective tissues around the epineurium, nerves were then transferred to fresh phosphate buffered saline (PBS) and preserved at 4°C until further use (for maximum of 24 h).

The obtained nerves were trimmed into 10 mm segments and randomly assigned to three groups as follows.

*Native nerves (Native)*: This group contains unprocessed sciatic nerves as a control for all analyses.

**FIGURE 1:**
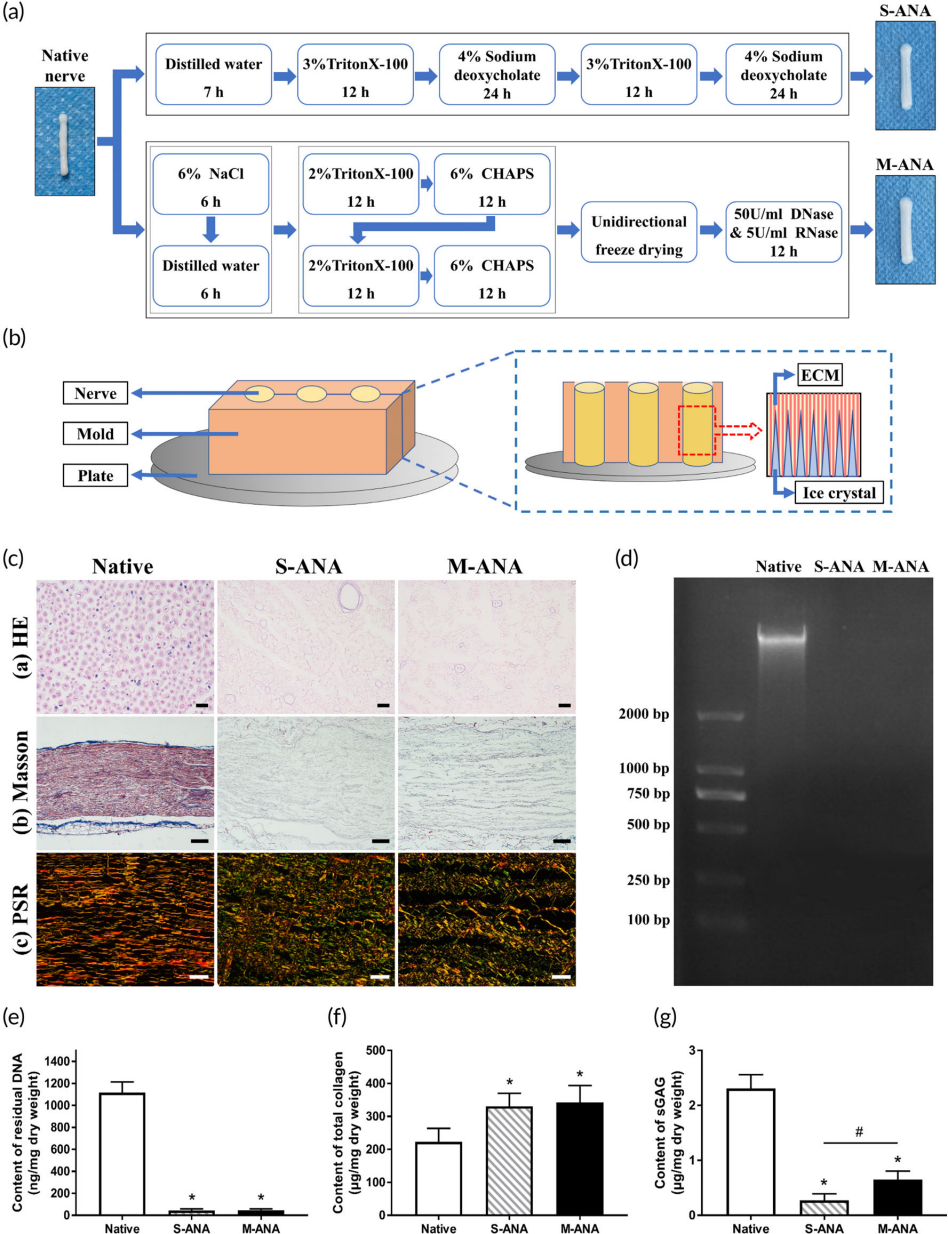
Fabrication of acellular nerve allografts and evaluation of decellularization extent and extracellular matrix (ECM) preservation. (a) The schematic diagram shows the decellularization method of multichannel acellular nerve allografts (M‐ANA) and Sondell acellular nerve allografts (S‐ANA). (b) The schematic diagram of the ultrastructural optimization principle of unidirectional freeze‐drying. The hematoxylin and eosin (HE) staining (ca, scale bar = 20 μm) was conducted to assess the removal of cell components. The Masson trichrome staining (cb, scale bar = 100 μm) and the Picrosirius Red (PSR) staining (cc, scale bar = 50 μm) revealed the collagen integrity and distribution after decellularization. The DNA remnants were analyzed further by agarose gel electrophoresis (d) and detection of DNA content (e). The detection of total collagen (f) and sulfated GAGs (g) illustrated the effects of different decellularization methods on ECM components. Data are expressed as the mean ± SD (*n* = 5). *p < 0.05 compared to native nerves, ^#^ *p* < 0.05 compared to S‐ANA.

*Sondell ANAs (S‐ANA)*: As a standard for comparison, the allografts in this group were generated using the decellularization method previously proposed by Sondell et al.^18^ (Figure 1a). Briefly, nerve segments were immersed in distilled water for 7 h, followed by two successive chemical extraction processes in 3% (v/v) Triton X‐100 (Sigma‐Aldrich, USA) for 12 h and 4% (w/v) SDC (Sigma‐Aldrich) for 24 h at room temperature under constant agitation (120 rpm).

*Multichannel ANAs (M‐ANA)*: This group contains the allografts decellularized by the modified method devised in our lab (Figure 1a).

The nerve segments were first incubated in 6% (w/v) sodium chloride for 12 h, followed by rinsing in distilled water for 12 h. Next, these segments were treated with 2% (v/v) Triton X‐100 for 12 h, followed by washing three times with distilled water (15 min each), then transferred to 6% (w/v) CHAPS (Sigma‐Aldrich) for 12 h incubation. This treatment was repeated with the two detergents. All procedures were at room temperature and under constant agitation (120 rpm).

After washing in distilled water (three times for 15 min each), unidirectional freeze‐drying was conducted to form optimized multichannel ultrastructure. In this step, a custom‐made silicon rubber mold and a stainless steel plate were applied. The mold serves to hold nerve segments perpendicular to the plate and insulate the surrounding heat transfer, thus ensuring the heat transfer along the long axis of nerves. The stainless steel plate was precooled (−80°C) to establish unidirectional temperature gradient inside the mold, which allows longitudinal ice crystals to form within nerves (Figure 1b). Briefly, the procedure is to first put nerve segments into the prefabricated grooves in one side of the mold, and then close the mold. Subsequently, the mold containing nerve segments was placed on the precooled stainless steel plate, and rapidly transferred to a freezer (−40°C). After a 1 h hold period, the mold was moved to a freeze dryer and lyophilized for 24 h to remove ice crystals and generate microchannels (Figure S1).

After rehydration in PBS overnight, the nerve segments were subjected to an enzymatic treatment with DNase (50 U/ml) and RNase (5 U/ml) at 37°C for 12 h under constant agitation (120 rpm).

After thoroughly washing in PBS (three times for 1 h each), the processed nerve segments of each group were stored in PBS at 4°C until use.

### 2.3 Histological analysis

The samples of both Native and ANAs were fixed in 4% paraformaldehyde, dehydrated with ethanol, hyalinized with xylene, embedded in paraffin, and cut into 5 μm sections. Transverse sections were stained with hematoxylin and eosin (HE) to assess the removal of cellular components. Masson trichrome staining and Picrosirius Red (PSR) were conducted in longitudinal sections to analyze the morphological and componential characterization of collagen fibers.

### 2.4 Biochemical analysis

Residual DNA content and size were measured to evaluate the extent of decellularization. Total DNA was extracted from samples using a genomic DNA extraction kit (Takara, Japan) and quantified using a NanoPhotometer N50 (IMPLEN, Germany). The size of the extracted DNA fragments was detected by 1% agarose gel electrophoresis.

The amount of collagen and sulfated glycosaminoglycans (GAGs) was measured to assess the abundance of ECM components. Total collagen content was determined by quantifying the hydroxyproline content with a hydroxyproline assay kit (Sigma‐Aldrich). Because hydroxyproline is largely restricted to collagen, it serves as an indicator for the amount of collagen in tissues. Total collagen content was calculated based on a hydroxyproline‐to‐collagen ratio of 1:7.69. In addition, sulfated GAGs content was quantified by 1,9‐dimethylmethylene blue dye‐binding assay using a Blyscan kit (Biocolor, UK).

Each procedure was conducted according to the manufacturer's instructions. All biochemical measurements were normalized to tissue dry weight and five samples were used for each group.

### 2.5 Western blot analysis

To evaluate the effects of different decellularization methods on the preservation of main ECM molecules, laminin, fibronectin, collagen I and IV in M‐ANA and S‐ANA were detected by immunoblotting. Total protein from samples was extracted and quantified using a BCA protein kit (Thermo Fisher Scientific, USA). Equal amounts of protein were separated by electrophoresis on two sodium dodecyl sulfate polyacrylamide gels. One gel was stained with Coomassie Brilliant Blue R‐250 to quantify total protein, serving as a control for loading. The other one was transferred to a PVDF membrane. After blocking, the membrane was incubated with the anti‐laminin, anti‐fibronectin, anti‐collagen I and IV antibody (1:1000, Abcam, USA) overnight at 4°C, followed by the second antibody (1:10000, Abcam) for 1 h at room temperature. Protein bands were visualized with an enhanced chemiluminescence kit (Thermo Fisher Scientific) and quantified by gray scale analysis using Image J (National Institutes of Health, USA). All detections were independently repeated three times.

### 2.6 Enzyme‐linked immunosorbent assay

To analyze the effects of different decellularization methods on the retention of critical regenerative factors, enzyme‐linked immunosorbent assays (ELISAs) were conducted to detect nerve growth factor (NGF), vascular endothelial growth factor (VEGF), and brain‐derived neurotrophic factor (BDNF) in M‐ANA and S‐ANA. ELISA kits (Thermo Fisher Scientific) were used according to the manufacturer's instructions. The concentrations of NGF, VEGF, and BDNF were calculated from the standard curve. All detections were independently repeated three times.

### 2.7 Scanning electron microscopy analysis

Scanning electron microscopy (SEM) was performed to reveal the ultrastructural features of each group. Samples were fixed in 2.5% glutaraldehyde for 2 h and postfixed in 1% osmic acid for 30 min at 4°C. Then, samples were quenched in liquid nitrogen and broken to achieve fracture surfaces. After dehydration through a gradient of ethanol and critical point drying, cross sections were sprayed with platinum and observed with an SEM (VEGA3, TESCAN, Czech). SEM images were used to characterize internal morphology and distribution of pore size in each group. Five samples for each group were analyzed and five random high power visual fields were selected in each sample, located in the upper, lower, middle, left, and right part of the cross sections. Over 200 pores per group were measured to obtain distribution and average of pore size. The pore size was measured by Image‐Pro Plus (Media Cybernetics, USA) and expressed as the mean of its shortest and longest diameters.

### 2.8 Porosity test

The porosity of each group was determined by the gas‐ethanol replacement method. Briefly, the lyophilized samples were immersed in definite volume (*V*_0_) of anhydrous ethanol in a graduated cylinder for 10 min, the total volume of the ethanol‐impregnated samples and ethanol was recorded as *V*_1_. Then, the samples were taken out and the volume of residual ethanol was recorded as *V*_2_. Five samples were examined for each group. The porosity was calculated by the following formula:

$$\text{Porosity}\;\left(\%\right)=\left(V_{0}-V_{1}\right)/\left(V_{1}-V_{2}\right)\times 100\%.$$

### 2.9 Swelling test

The swelling behavior of each group was tested using a rehydration study. The samples were first lyophilized to obtain a constant dry weight (*W*_0_). Then, the lyophilized samples were immersed in PBS for 24 h at room temperature. After taking the samples out of PBS, the excess water was removed by filter papers from the surface to achieve a constant swelling weight (*W*_s_). Five samples were examined for each group. The swelling ratio was calculated by the following formula:

$$\text{Swelling ratio}=\left(W_{s}-W_{0}\right)/W_{0}\times 100\%.$$

### 2.10 Biomechanical test

To assess the biomechanical properties of each group, a tensile test was carried out using a dynamic electromechanical testing instrument (Tianjin, China). Tensile uniaxial stress was applied parallel to the longitudinal axis of samples at a constant strain rate of 10 mm/min. Samples were kept moist during testing. Five samples were examined for each group.

To detect tensile properties, both ends of samples were mounted on custom clamps fitted with sandpapers, leaving a distance of 10 mm between the clamps. Each sample was stretched to complete tensile failure. Young's modulus, stress at fracture, and strain at fracture were measured.

To measure suture retention strength, every sample was sutured between two stumps of fresh rat sciatic nerves with 8–0 nylon sutures. The suture was pierced through epineurium at 1 mm from the edge and knotted at least seven times to ensure no slippage. The other side of the fresh nerve was clamped into the testing instrument. Suture retention strength was defined as the maximum load when sutures were pulled out of epineurium.

### 2.11 Biocompatibility evaluation

#### 2.11.1 In vitro study for penetration of Schwann cells

To evaluate the ability of M‐ANA and S‐ANA to support Schwann cell infiltration and migration, a cell penetration assay was conducted. Samples were preimmersed in DMEM/F‐12 containing 10% FBS and 1% penicillin/streptomycin at 37°C for 24 h. The extracted primary Schwann cells (Figure 5a) were resuspended in the medium at a concentration of 1 × 10^7^ cells/ml. Then, 5 μl of the cell suspension was seeded on one end of the samples in six‐well plates at a constant speed (0.5 μl/s). After 30 min, another 5 μl of the cell suspension was seeded on the same end of the samples. After 1 h, 2.5 ml medium was added to every well. The samples seeded with Schwann cells were cultured for 7 days. Thereafter, 10‐μm longitudinal sections of each sample were prepared and stained with 4′,6‐diamidino‐2‐phenylindole dihydrochloride (Sigma‐Aldrich) to mark the nuclei of Schwann cells for analyzing the proliferation and penetration behavior of Schwann cells within different ANAs in vitro.

#### 2.11.2 Detection of DNA content in culture medium

The DNA content in culture medium was detected to evaluate the extent of cell damage, thereby analyzing the biocompatibility of M‐ANA and S‐ANA. The DNA content in the 3‐day culture medium of the cell penetration assays was quantified by a NanoPhotometer N50 (IMPLEN). Schwann cells cultured without any sample served as a negative control, and Schwann cells incubated with 2% Triton X‐100 served as a positive control.

#### 2.11.3 Cytotoxicity evaluation

Cell Counting Kit‐8 (CCK‐8) was used to assess the cytotoxicity of M‐ANA and S‐ANA. Extract medium was prepared by incubating 0.1 g sample of each group in 1 ml DMEM at 37°C for 72 h. Moreover, a normal medium control (DMEM), a negative control (polyethylene), a positive control (DMSO), and a blank control (DMEM without cells) were prepared. RSC96 cells were seeded into 96‐well plates at a concentration of 1 × 10^4^ cells/well. After refreshing medium, 10 μl CCK‐8 solution was added to each well, followed by incubation for 2 h. The absorbance was measured at 450 nm. Cell proliferation was assessed at 24, 48, and 72 h, respectively. Five samples for each group were analyzed at each time point.

### 2.12 Real‐time polymerase chain reaction

Real‐time polymerase chain reaction (RT‐PCR) was conducted to analyze the effects of M‐ANA and S‐ANA on the gene expressions of myelin protein zero (MPZ) and NGF Receptor p75 (NGFR p75) in Schwann cells. For in vitro culture of Schwann cells, the 10‐μm cryosections of M‐ANA and S‐ANA were prepared and collected on glass coverslips. After OCT removal and sterilization with 75% alcohol, the coverslips with the graft membranes were completely washed with PBS and placed in six‐well plates. The primary Schwann cell suspension was seeded to each well at a concentration of 2 × 10^5^ cells/well. The cells cultured on blank coverslips served as the control group. After incubation for 3 days, total RNA was extracted from the Schwann cells in each group using TRIzol reagent (Invitrogen, USA). Then, 1 μg of total RNA was reversely transcribed into complementary DNA (cDNA) using a PrimeScript RT reagent kit (Takara) according to the manufacturer's instructions. RT‐PCR was performed in the Light Cycler RT‐PCR Detection System (Roche, Switzerland) using TB Green Premix Ex Taq II (Takara). Then, 1 μl of cDNA was used for PCR amplification in a final volume of 10 μl. The PCR amplification reaction was performed under the following conditions: 3 min at 95°C for initial denaturation, followed by 40 cycles of 30 s at 95°C for denaturation, 30 s at 60°C for annealing, and 45 s at 72°C for extension. The relative expression level of each target gene was calculated using the 2^−ΔΔC^ ^t^ method and normalized to the level of GAPDH mRNA. The primer sequences are listed in Table S1. All detections were independently repeated three times.

### 2.13 In vivo study for functionalities

#### 2.13.1 Surgical procedures

Male Sprague Dawley rats (*n* = 48) were randomly divided into three groups: autologous nerve graft (ANG), S‐ANA, and M‐ANA. All surgical procedures were performed by the same surgeon under aseptic condition with sodium pentobarbital anesthesia (intraperitoneal, 40 mg/kg body weight). An incision in the skin was made at the level of the right biceps femoris, the muscle was then dissected along the connective fascia to expose the sciatic nerve. An 8 mm segment of sciatic nerve was excised, leaving a 10 mm defect after retraction of nerve stumps. For ANG, the transected nerve segment was reversed and sutured back into the gap. For S‐ANA and M‐ANA, the ANA was sutured to the epineurium of the two nerve stumps. All operations were performed using 8–0 nylon sutures under a surgical microscope. Then, the muscle and skin were closed using standard techniques. Except for gait analysis (2, 4, 6, 8, 10, and 12 weeks), other assessments were performed at 6 and 12 weeks postimplantation, respectively (*n* = 8 per group at each time point).

#### 2.13.2 Gait analysis

Gait analysis was performed to evaluate motor functional recovery. After dipping their hind feet in ink, the rats of each group were placed in a clear corridor with a dark shelter at the end. Paw length (PL), toe spread (TS), and intermediary TS (ITS) of the normal (N) and experimental (E) feet were measured. The sciatic functional index (SFI) was calculated according to the following formula^34^:

$$\mathrm{SFI}=-38.3\left[\left(\mathrm{EPL}-\mathrm{NPL}\right)/\mathrm{NPL}\right]+109.5\left[\left(\mathrm{ETS}-\mathrm{NTS}\right)/\mathrm{NTS}\right]+13.3\left[\left(\text{EITS}-\text{NITS}\right)/\text{NITS}\right]-8.8.$$

#### 2.13.3 Electrophysiological analysis

Electrophysiological analysis was conducted to assess nerve functional recovery by the measurement of compound muscle action potentials (CMAPs). The surgical sites of sciatic nerves were exposed under anesthesia. The electrical stimuli (stimulus intensity = 6 mA; frequency = 1 Hz; duratio*n* = 1 ms) were applied to the proximal and distal ends of each graft sequentially, and the CMAPs of ipsilateral anterior tibial muscle were recorded. CMAP amplitude was expressed as the ratio of surgical side to normal side. Motor nerve conduction velocity (MCV) was calculated based on latency and the distance between the two stimulating sites.

#### 2.13.4 Muscle wet weight evaluation

After the electrophysiological test, the rats were euthanized by sodium pentobarbital overdose. Then, the bilateral anterior tibial muscles of each group were carefully dissected and immediately weighed to evaluate muscle recovery. The ratio of muscle wet weight was expressed as the percentage of surgical side to normal side.

#### 2.13.5 Toluidine blue staining

Toluidine blue staining was performed to assess newborn axon myelination. The nerve segments of central grafts and distal stumps were fixed in 2.5% glutaraldehyde, followed by postfixation in 1% osmium tetroxide. After dehydration in a graded series of ethanol, the processed segments were embedded in Epon 812 epoxy resin. Transverse semi‐thin sections of central grafts and distal stumps were cut using an ultramicrotome and stained with toluidine blue. The stained sections were observed under a light microscope (BX53, Olympus, Japan), and five random fields were selected for each semi‐thin section to determine the density of myelinated axons by Image J.

#### 2.13.6 Transmission electron microscope analysis

Transmission electron microscopy (TEM) was used to analyze the micromorphological characteristics of nerve fibers growing into distal stumps, thereby further evaluating their remyelination extent. For TEM observation, transverse ultrathin sections of distal stumps were prepared and stained with uranyl acetate and lead citrate, and then observed under a TEM (HT7700, Hitachi, Japan). Images were acquired from 10 random fields of each ultrathin section and analyzed with Image J to measure the mean thickness of myelin sheaths and the mean diameter of myelinated nerve fibers. G‐ratio was calculated as the ratio of the axon diameter (fiber minus myelin sheath) to the fiber diameter.

#### 2.13.7 Immunofluorescence staining

Immunofluorescence staining was performed to further analyze axons and myelin sheaths of regenerative nerves. After fixation and dehydration, the nerve segments of central grafts and distal stumps were embedded in OCT and cut using a freezing microtome (CM1900, Leica, Germany) to prepare 10‐μm transverse sections. The sections were blocked with normal goat serum, and treated with anti‐neurofilament 200 antibody (NF200, 1:100, Abcam) and anti‐S‐100 antibody (1:200, Abcam) overnight at 4°C, followed by incubation with fluorescent‐conjugated secondary antibodies (1:1000, Abcam) for 1 h at 37°C. The stained sections were imaged using a laser scanning confocal microscopy (FV1200, Olympus, Japan). The percentage of NF200‐positive area within selected fields was measured with Image‐Pro Plus (Media Cybernetics).

### 2.14 Statistical analysis

Data were presented as mean ± standard deviation (SD) and were statistically analyzed using GraphPad Prism 5.0 (GraphPad Software, USA). Differences between two groups were analyzed with Student's *t* test, and differences among three or more groups were analyzed with one‐way or two‐way ANOVA followed by Tukey's post hoc test for multiple comparisons. A value of *p* < 0.05 was considered statistically significant.

## 3 RESULTS

### 3.1 Decellularization extent evaluation

The decellularization extent of ANAs was evaluated by histological and biochemical analysis. The HE staining showed that, as with S‐ANA, there were no visible cell nuclei in M‐ANA (Figure 1c). The DNA quantification demonstrated that, after the modified decellularization treatment, the DNA content of M‐ANA exhibited a remarkable decrease compared to unprocessed Native (*p* < 0.05), which is comparable to S‐ANA (*p* > 0.05) (Figure 1e). Moreover, in the agarose gel electrophoresis, no obvious band of DNA fragments was found in M‐ANA (Figure 1d).

### 3.2 ECM morphological characterization

The ECM morphology of ANAs was characterized by Masson trichrome staining, PSR staining, and SEM (Figures 1c and 3a). Native had the typical organization of endoneurium that wrapped axons and myelin sheaths. Longitudinally oriented collagen fibers that were tight and neat can be observed within Native. These fibers were mainly composed of Collagen I with a few scattered Collagen III. After decellularization, axons and myelin sheaths were effectively eliminated in M‐ANA and S‐ANA. S‐ANA preserved the compact ultrastructure of basal lamina tubes, but the arrangement of collagen fibers was disorganized. Furthermore, in addition to Collagen I, S‐ANA exposed more Collagen III after decellularization. In M‐ANA, it was observed the presence of the optimized multichannel ultrastructure. The collagen fibers exhibited a loose distribution. There were many continuous microchannels aligned axially between parallel fibers. Similar to S‐ANA, a large amount of Collagen III can be seen uniformly distributed around Collagen I in M‐ANA.

### 3.3 ECM componential characterization

To assess the remaining ECM components after decellularization, this study has quantitatively examined the amount of total collagen and sulfated GAGs in Native and ANAs. The total collagen contents in M‐ANA and S‐ANA increased significantly compared to Native following processing (*p* < 0.05), but they were not significantly different from each other (*p* > 0.05) (Figure 1f). In terms of sulfated GAGs content, M‐ANA and S‐ANA exhibited a significant decrease in comparison with Native (*p* < 0.05). M‐ANA had significantly more sulfated GAGs than S‐ANA (*p* < 0.05) (Figure 1g).

**FIGURE 2:**
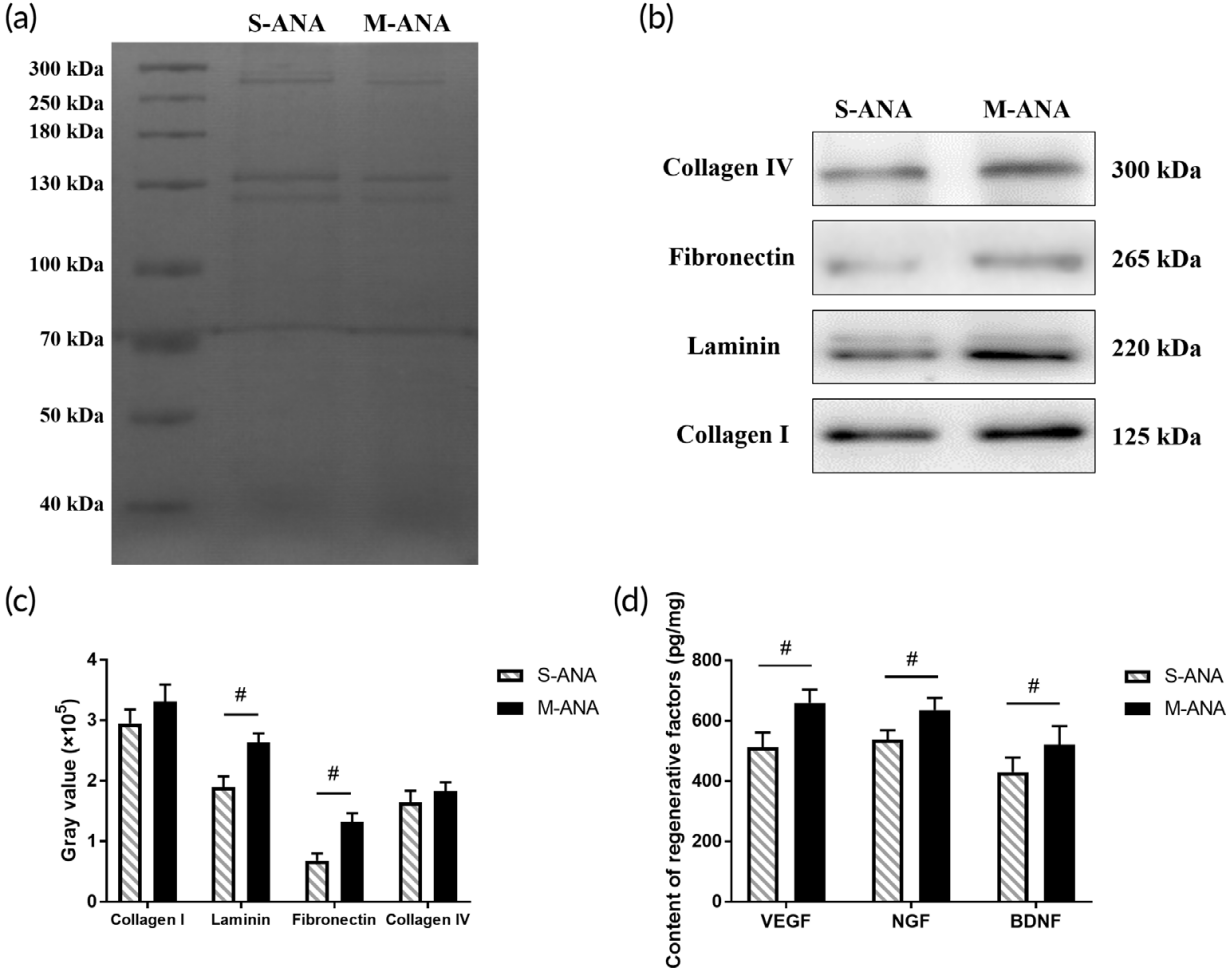
Comparison of main extracellular matrix (ECM) bioactive molecules and regenerative factors in multichannel acellular nerve allografts (M‐ANA) and Sondell acellular nerve allografts (S‐ANA). (a) Total protein in the two groups was detected by Coomassie blue staining and used as a control for loading. (b,c) Fibronectin, laminin, Collagen I and IV were determined by Western blot and quantified by gray scale analysis. (d) Vascular endothelial growth factor (VEGF), nerve growth factor (NGF), and brain‐derived neurotrophic factor (BDNF) were detected by enzyme‐linked immunosorbent assay (ELISA). Data are expressed as the mean ± SD (*n* = 5). ^#^ *p* < 0.05 compared to S‐ANA.

To further analyze the ability of different decellularization methods to preserve desirable matrix proteins, the amounts of critical bioactive molecules and factors in two acellular groups were compared using Western blot and ELISA, respectively. Western blot showed that the levels of Collagen I and IV retained in M‐ANA and S‐ANA were comparable (*p* > 0.05). But there were significantly higher levels of laminin and fibronectin in M‐ANA than in S‐ANA (*p* < 0.05) (Figure 2a–c). Moreover, ELISA revealed that M‐ANA had significantly higher contents of VEGF, NGF, and BDNF compared to S‐ANA (*p* < 0.05) (Figure 2d).

### 3.4 Pore size and porosity analysis

**FIGURE 3:**
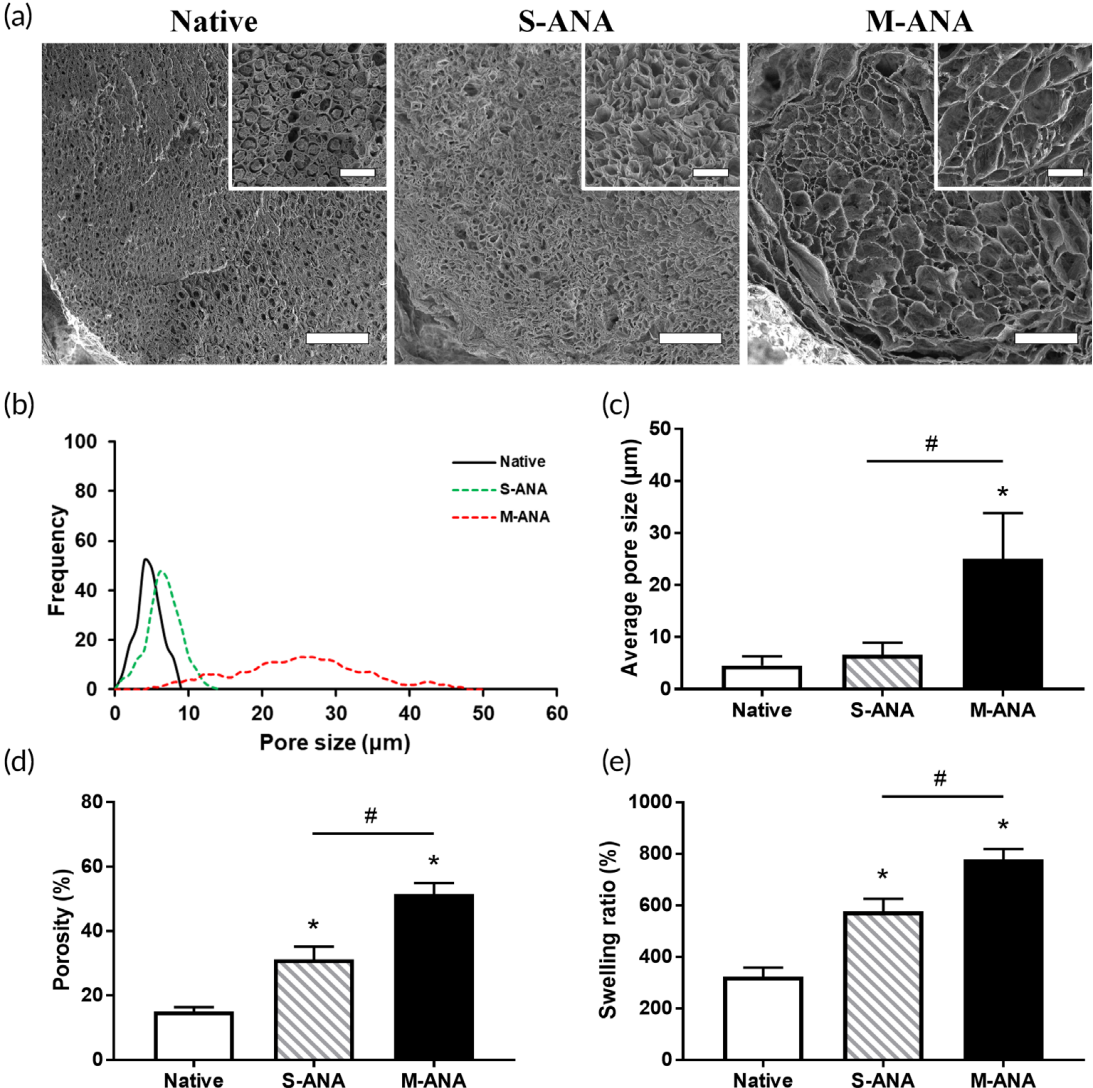
Analyses of pores and swelling behavior. (a) The scanning electron microscopy (SEM) images of transverse sections of native nerves, multichannel acellular nerve allografts (M‐ANA), and Sondell acellular nerve allografts (S‐ANA) at ×700 magnification (scale bar = 50 μm). The insets were the SEM images at ×2000 magnification (scale bar = 20 μm). Frequency distribution of pore sizes (b), average pore size (c), and porosity (d) were assessed to investigate the characteristics of pores. Moreover, swelling behavior analysis was performed to assess the effects of ultrastructure on internal liquid exchange (e). Data are expressed as the mean ± SD. *p < 0.05 compared to native nerves, ^#^ *p* < 0.05 compared to S‐ANA.

Pore size analysis and porosity test were performed to determine the ultrastructural characterization of ANAs. The distribution of pore size was assessed according to SEM. After decellularization, S‐ANA showed an obvious increase in average pore size compared to Native (*p* < 0.05), but the majority of pore sizes were still below 10 μm. However, the pore size of M‐ANA was mainly distributed between 20 and 35 μm, with average pore size of 25.08 ± 8.75 μm, which was significantly larger than the other groups (*p* < 0.05) (Figure 3b,c). In addition, the results of the porosity test suggested that the porosity of M‐ANA and S‐ANA increased significantly compared to that of Native following decellularization (*p* < 0.05). M‐ANA exhibited significantly higher porosity than S‐ANA (*p* < 0.05) (Figure 3d).

### 3.5 Swelling characterization

The swelling characterization was analyzed to assess the ability of water absorption. In comparison with Native, M‐ANA and S‐ANA achieved a significantly higher swelling ratio following processing (*p* < 0.05). Moreover, the swelling ratio was remarkably higher in M‐ANA than in S‐ANA (*p* < 0.05) (Figure 3e).

### 3.6 Biomechanical properties

**FIGURE 4:**
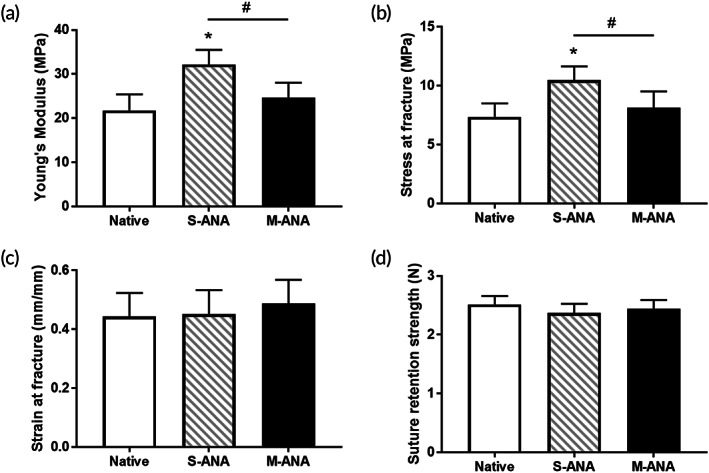
Evaluation of biomechanical properties. Young's modulus (a), stress at fracture (b), strain at fracture (c), and suture retention strength (d) were evaluated to analyze biomechanical properties of native nerves, Sondell acellular nerve allografts (S‐ANA), and multichannel acellular nerve allografts (M‐ANA). Data are expressed as the mean ± SD (*n* = 5). *p < 0.05 compared to native nerves, ^#^ *p* < 0.05 compared to S‐ANA.

The biomechanical test was conducted to determine the changing properties of ANAs following different decellularization processes. The Young's modulus and stress at fracture in M‐ANA were not markedly different from those in Native (*p* > 0.05). However, S‐ANA exhibited a significant increase compared to Native (*p* < 0.05), and was significantly higher than M‐ANA (*p* < 0.05). In addition, there was no significant difference in the strain at fracture and suture retention strength among the three groups (*p* > 0.05) (Figure 4).

### 3.7 Biocompatibility evaluation

#### 3.7.1 Schwann cell penetration analysis

**FIGURE 5:**
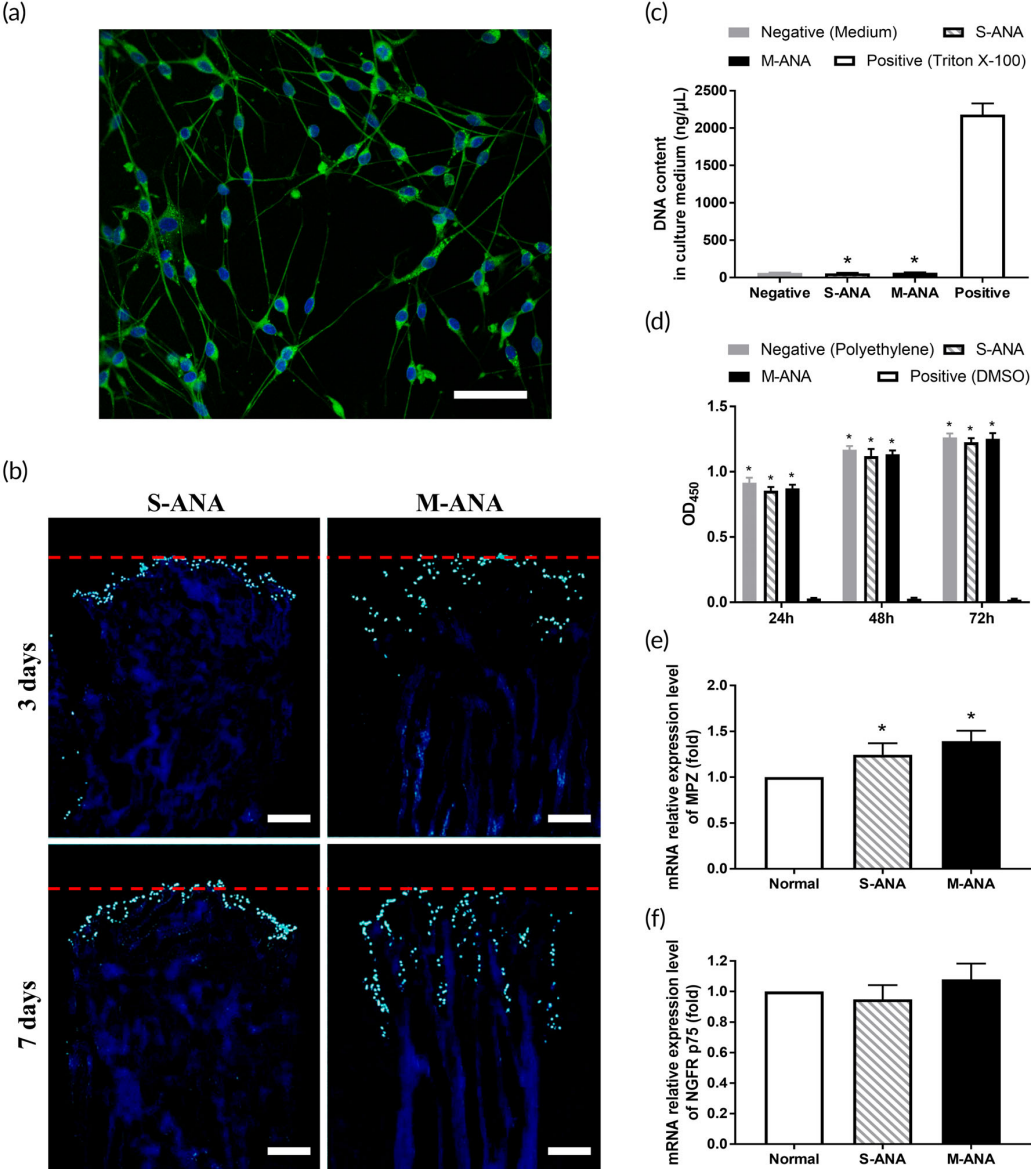
Comparison of ability to support penetration of Schwann cells and biocompatibility of multichannel acellular nerve allografts (M‐ANA) and Sondell acellular nerve allografts (S‐ANA). (a) Primary Schwann cells were identified by S‐100 (green) immunofluorescence staining (scale bar = 50 μm). It was observed that the extracted Schwann cells were positive for S‐100 and exhibited the typical morphology of bipolar spindle shape. (b) The 4′,6‐diamidino‐2‐phenylindole dihydrochloride (DAPI) staining of longitudinal sections revealed the proliferation and penetration of Schwann cells in S‐ANA and M‐ANA at 3 and 7 days after seeding (scale bar = 100 μm). The labeled nuclei were shown in bright blue. The red dashed lines represent the initial seeding sites. The detection of DNA content in the medium (c) and CCK‐8 assay of extract medium (d) were performed to analyze the cytotoxicity of M‐ANA and S‐ANA. The gene expressions of myelin protein zero (MPZ) (e) and nerve growth factor receptor p75 (NGFR p75) (f) in Schwann cells were detected to analyze the effects of M‐ANA and S‐ANA on cellular function. Data are expressed as the mean ± SD. *p < 0.05 compared to positive/normal control.

The penetration behavior of Schwann cells was analyzed to determine the effects of optimized ultrastructure on the functional characteristics of ANAs. After culturing for 3 days, no significant cell penetration into ANAs was observed in S‐ANA and the majority of cells were still located in the inoculating position. By contrast, through the internal microchannels of M‐ANA, numerous cells have infiltrated into the samples. After culturing for 7 days, Schwann cells in both groups exhibited remarkable proliferation. Most cells in S‐ANA remained in the superficial area of the samples. Nevertheless, for M‐ANA, large numbers of cells were found to proliferate and migrate further along the microchannels (Figure 5b).

#### 3.7.2 DNA release detection

DNA released from damaged or dead cells was quantified to evaluate the biocompatibility of ANAs. It was found that, as with the negative control group, almost no DNA release could be detected in the medium of both M‐ANA and S‐ANA (*p* > 0.05). Moreover, their DNA contents were far lower than that of the positive control group (*p* < 0.05) (Figure 5c).

#### 3.7.3 Cytotoxicity analysis

CCK‐8 was performed at 24, 48, and 72 h as a complement to provide a thorough assessment of biocompatibility. The results showed that the cells in each group proliferated with time. At three time points, the OD_450_ values of M‐ANA and S‐ANA were comparable (*p* > 0.05) and exhibited no significant difference compared to that of the negative control group (*p* > 0.05). Moreover, they were significantly higher than the positive control group (*p* < 0.05) (Figure 5d).

#### 3.7.4 Gene expression analysis

MPZ and NGFR p75 are closely associated with the myelination of newborn axons. Their gene expressions in Schwann cells were detected by RT‐PCR. The results showed that the mRNA relative expression levels of MPZ were significantly upregulated in M‐ANA and S‐ANA (*p* < 0.05). M‐ANA appeared to be higher than S‐ANA, but no statistical difference was observed (*p* > 0.05) (Figure 5e). The relative expression level of NGFR p75 showed a slight upregulation in M‐ANA (*p* > 0.05). The minor downregulation of NGFR p75 gene expression was detected in S‐ANA (*p* > 0.05). However, there was no significant difference between M‐ANA and S‐ANA (*p* > 0.05) (Figure 5f).

### 3.8 In vivo study

#### 3.8.1 Motor function evaluation

**FIGURE 6:**
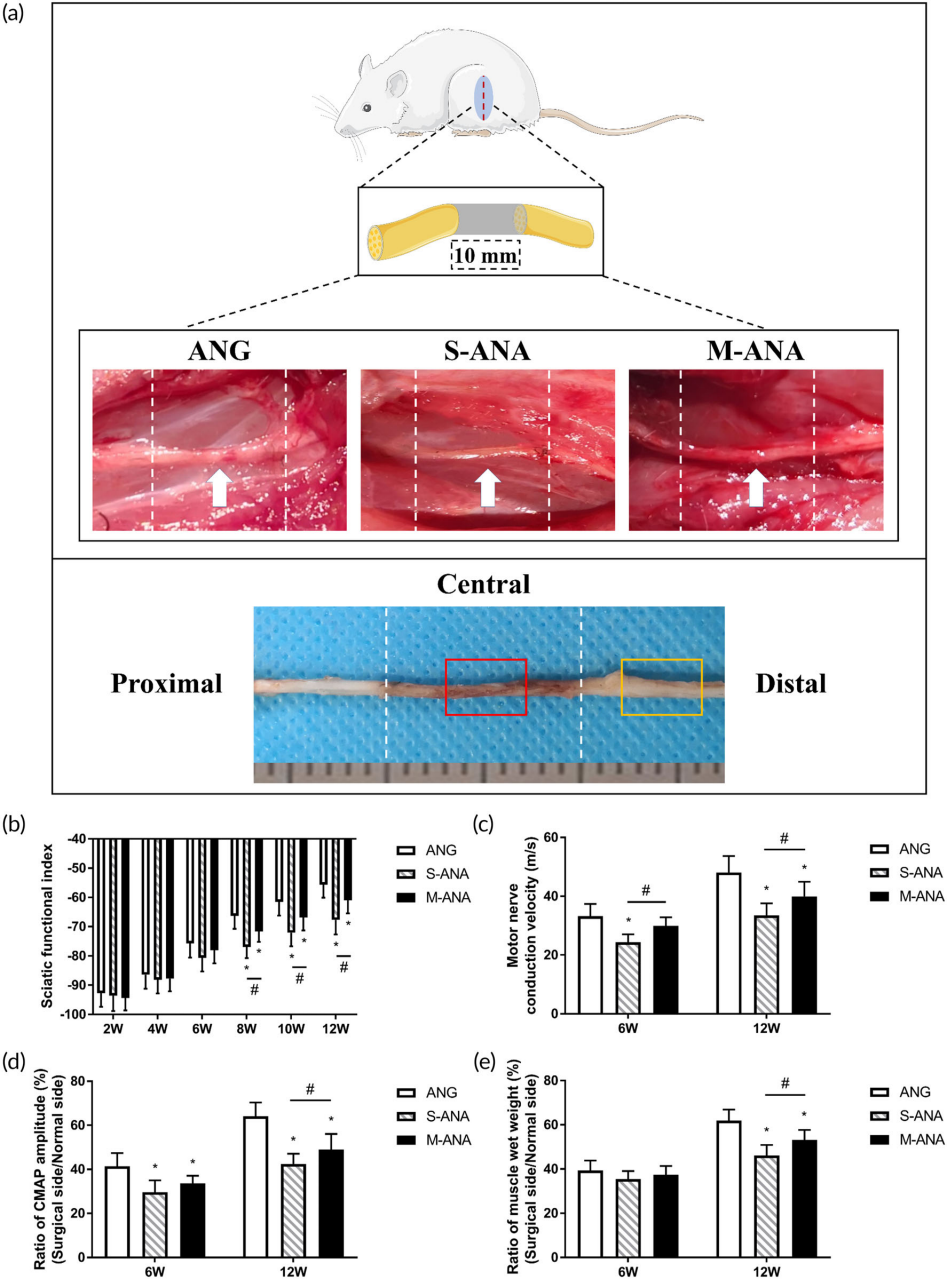
The schematic diagram of the in vivo study and evaluation of functional recovery and electrophysiology. (a) The schematic diagram of the in vivo study. The red and orange boxes represent the sampling location of the central grafts and distal stumps, respectively. The histograms show sciatic functional index (SFI) values at 2, 4, 6, 8, 10, and 12 weeks after transplantation (b), and motor nerve conduction velocities (MCVs) (c), ratios of CMAP amplitude (d), and ratios of muscle wet weight (e) at 6 and 12 weeks after transplantation. Data are expressed as the mean ± SD (*n* = 8). *p < 0.05 compared to ANG, ^#^ *p* < 0.05 compared to Sondell acellular nerve allografts (S‐ANA).

SFI is a key indicator to assess the motor functional recovery following repair. It was found that each group showed an improvement in SFI with time. However, no obvious difference among ANG, S‐ANA, and M‐ANA was observed over the first 6 weeks (*p* > 0.05). From 8 weeks, M‐ANA exhibited higher SFI than S‐ANA (*p* < 0.05), but was still significantly lower compared to ANG (*p* < 0.05) (Figure 6b).

#### 3.8.2 Electrophysiological analysis

Electrophysiological analysis allows for a meaningful assessment of the effective reinnervation of grafts able to deliver electrical stimuli to the target organs. At 6 weeks, the MCV of M‐ANA was significantly superior to that of S‐ANA (*p* < 0.05), and showed no significant difference compared to ANG (*p* > 0.05) (Figure 6c). In terms of CMAP amplitude, there was no obvious difference between M‐ANA and S‐ANA (*p* > 0.05), and they were significantly lower than ANG (*p* < 0.05). At 12 weeks, the MCV and CMAP amplitude of M‐ANA were remarkably greater than those of S‐ANA (*p* < 0.05), but were still inferior to those of ANG (*p* < 0.05) (Figure 6d).

#### 3.8.3 Muscle recovery evaluation

The wet weight ratio of anterior tibial muscle reflects the degree of muscle recovery after repair. The results showed that the wet weight ratios of all groups increased over time. At 6 weeks, there was no significant difference among the three groups (*p* > 0.05). At 12 weeks, M‐ANA had a higher wet weight ratio compared to S‐ANA (*p* < 0.05), but was significantly lower than ANG (*p* < 0.05) (Figure 6e).

#### 3.8.4 Analysis of axon regrowth and remyelination

**FIGURE 7:**
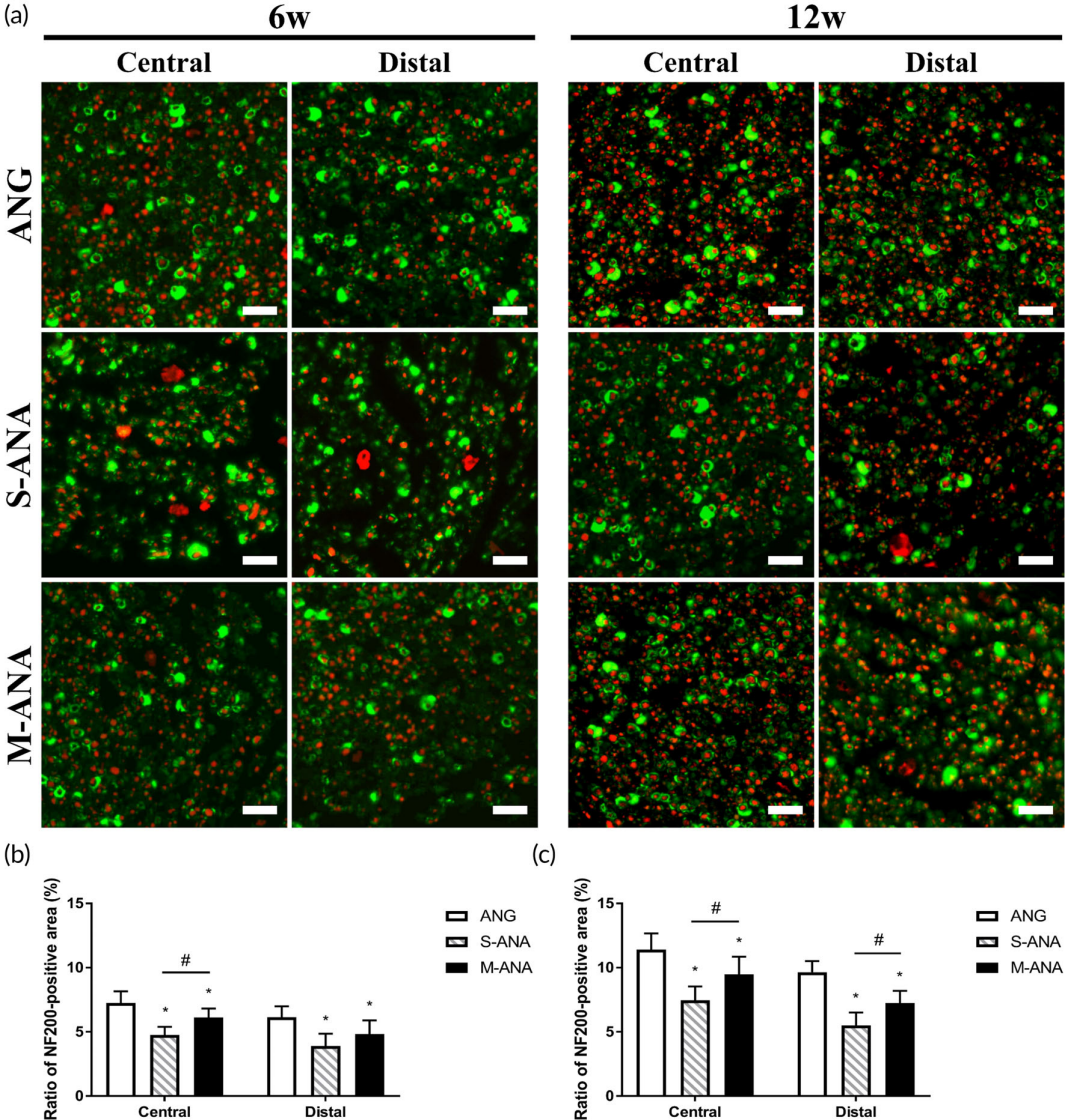
Evaluation of regenerative neurofilaments at 6 and 12 weeks after transplantation. (a) Immunofluorescence staining of NF200 (red) and S‐100 (green) in regenerative nerves (scale bar = 20 μm). The histograms show ratios of NF200‐positive area at 6 (b) and 12 weeks (c). Data are expressed as the mean ± SD (*n* = 8). *p < 0.05 compared to ANG, ^#^ *p* < 0.05 compared to Sondell acellular nerve allografts (S‐ANA).

Axon regrowth in newborn nerves was analyzed by immunofluorescence detection labeled with NF200 and S‐100 (Figure 7a). At 6 weeks, there was a significantly higher ratio of NF200‐positive area in M‐ANA compared to S‐ANA at the central grafts (*p* < 0.05). Moreover, at the distal stumps, M‐ANA also showed a tendency to a higher ratio, however, without statistical significance (*p* > 0.05) (Figure 7b). At 12 weeks, the ratios of both the central grafts and distal stumps in M‐ANA were significantly higher than those in S‐ANA (*p* < 0.05). At two time points, ANG exhibited the highest ratios at the central grafts and distal stumps (*p* < 0.05) (Figure 7c).

**FIGURE 8:**
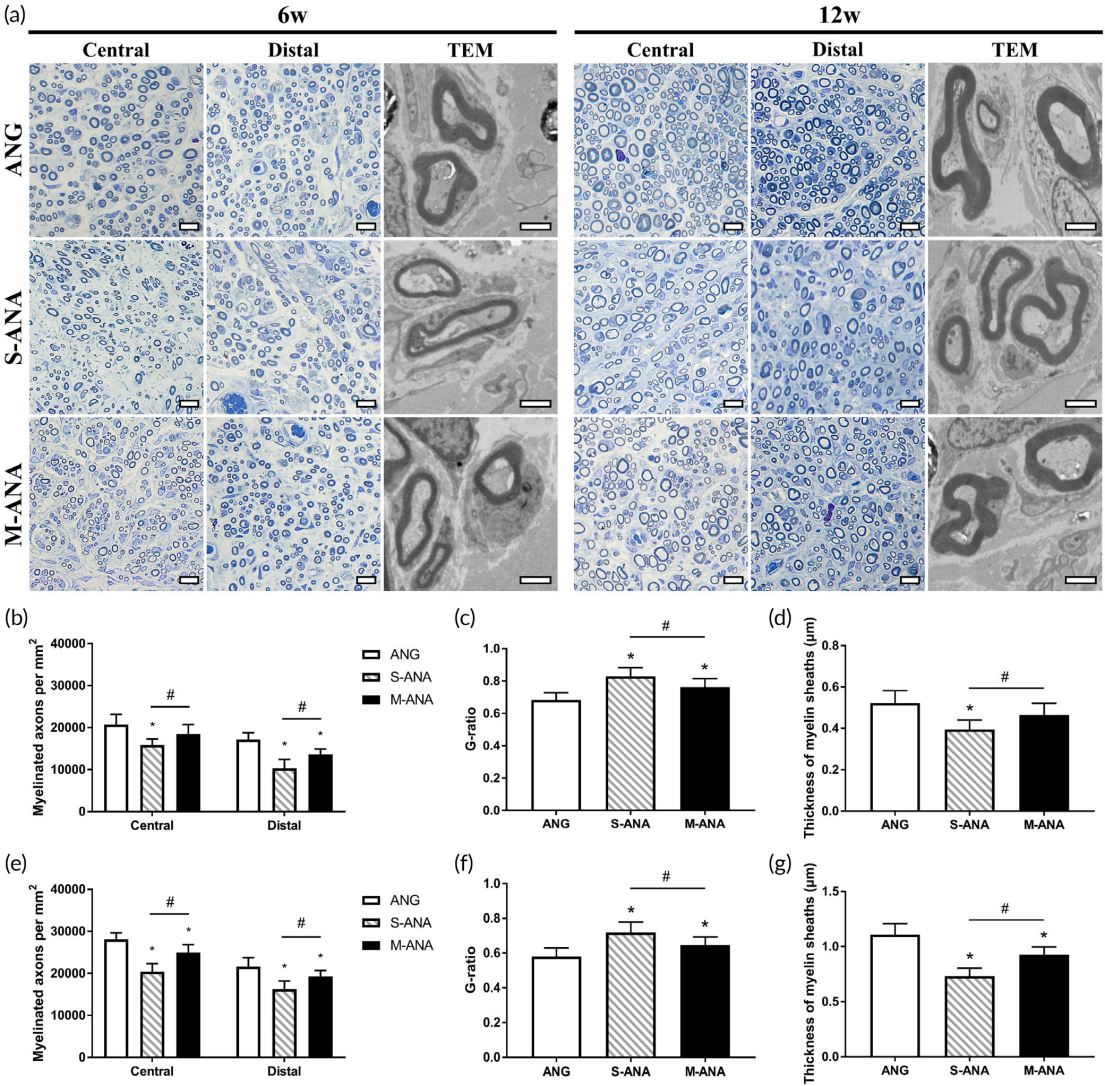
Evaluation of myelination of regenerative axons at 6 and 12 weeks after transplantation. (a) Toluidine blue staining (scale bar = 10 μm) and transmission electron microscopy (TEM) analysis (scale bar = 2 μm) of myelin sheaths in regenerative nerves. The histograms show densities of myelinated axons, G‐ratios, and thickness of myelin sheaths at 6 (b–d) and 12 weeks (e–g). Data are expressed as the mean ± SD (*n* = 8). *p < 0.05 compared to ANG, ^#^ *p* < 0.05 compared to Sondell acellular nerve allografts (S‐ANA).

Remyelination of regenerative nerves was evaluated by toluidine blue staining and TEM (Figure 8a). At 6 weeks, the myelinated axon densities of M‐ANA were significantly higher than S‐ANA at the central grafts and distal stumps (*p* < 0.05). Compared to ANG, M‐ANA showed no significant difference at the central grafts (*p* > 0.05). However, at the distal stumps, there was a significantly lower density in M‐ANA than in ANG (*p* < 0.05). At 12 weeks, at the central grafts and distal stumps, M‐ANA had significantly higher densities of myelinated axons compared to S‐ANA (*p* < 0.05), but was significantly inferior to ANG (*p* < 0.05) (Figure 8b,e). Assessment of axonal myelination by measuring the G‐ratio showed the lowest G‐ratio was obtained by ANG, closer to the optimal value of 0.6, with all other groups having significantly higher G‐ratios at both 6 and 12 weeks (*p* < 0.05). Notably, compared to S‐ANA, M‐ANA exhibited a lower G‐ratio (*p* < 0.05), which more closely resembled ANG (Figure 8c,f). In terms of myelin sheath thickness, at 6 weeks, M‐ANA was significantly thicker than S‐ANA (*p* < 0.05). The mean thickness of M‐ANA was slightly smaller than that of ANG, but the difference was not statistically significant (*p* > 0.05). At 12 weeks, M‐ANA was still significantly thicker than S‐ANA (*p* < 0.05), but remarkably smaller compared to ANG (*p* < 0.05) (Figure 8d,g).

## 4 DISCUSSION

ANAs are considered the potentially ideal nerve grafts for PNI treatment due to the preservation of ECM components and structure, as well as low immunogenicity.^35^ However, many current decellularization methods rely on harsh detergents to extract cellular antigens, which are destructive to the tissue matrix.^36^ Furthermore, the retained inherent neural structure composed of basal lamina tubes features small pore size and low porosity, which may have detrimental effects on nerve repair, whereas conventional decellularization protocols are incompetent to modify the ultrastructure of ANAs.^37^

To overcome the limitations of conventional nerve allografts, this study prepared a novel multichannel nerve allograft by our newly developed protocol consisting of multiple decellularization approaches. In detail, the incubations of hyper‐ and hypotonic solutions were performed first for the preliminary lysis of cells. Hypertonic solution can separate DNA from proteins and hypotonic solution can readily induce cell lysis with few deleterious effects on ECM.^38^ Then, repeated treatments with mild detergents, Triton X‐100 and CHAPS, were carried out to further lyse residual cells. Triton X‐100 can dissolve lipids on cell membranes, nuclear membranes, and organelle membranes by disrupting lipid–lipid and lipid–protein interactions, while leaving protein–protein interactions intact.^39^ CHAPS has a net zero electrical charge on the hydrophilic head groups, which is conducive to protecting the native state of ECM proteins during decellularization.^40^ These mild chemical approaches allow for the balance between the elimination of cellular antigens and the maintenance of ECM. Moreover, unidirectional freeze‐drying was utilized to improve the ultrastructure of ANAs. During unidirectional freeze‐drying process, parallel aligned ice crystals form and extend within allografts. Collagen fibers are compressed and concentrated between the growing ice crystals, thereby forming oriented microchannels, which imparts loose ultrastructure to ANAs. Notably, the optimized multichannel ultrastructure created by unidirectional freeze‐drying is more conducive to the infiltration of reagents in the subsequent step, while also providing favorable conditions for the effective clearance of the debris formed by cell lysis from tissues. Not only that, snap freezing can also lead to the formation of numerous intracellular ice crystals, which could cause damage to the structure and organization of cytoplasm, organelle membranes, and cell membranes, eventually destroying cells by direct mechanical action.^38^ In addition, drying can result in the removal of water bound to structural and functional biomolecules in the cells, which may affect cell stability and cause cell destruction.^41^ Therefore, the freeze‐drying served as a complement to the chemical treatments to further ensure adequate cell lysis in allografts, especially in the central parts where detergents cannot easily penetrate. At last, the treatment with DNase and RNase was conducted to further remove nucleotides and elute cellular debris within tissues. These nucleases can cleave nucleic acid sequences by catalyzing the hydrolysis of the phosphodiester bonds in the ribonucleotide or deoxyribonucleotide chains, which results in the degradation of RNA or DNA, facilitating the removal of these molecules from tissues.^42^ The combination of these techniques intends to optimize the internal ultrastructure of ANAs, and retain more ECM molecules and regenerative factors with removing cellular components as much as possible, thereby strengthening the neurorestorative capacity of ANAs.

The immunogenicity of allogeneic nerves is mainly derived from cells. The immunogenicity of neural ECM is extremely low or even negligible.^43^ The effective clearance of cellular components is a requisite for the preparation of ANAs. In this study, our new protocol achieved excellent decellularization outcomes similar to the Sondell method. M‐ANA exhibited an absence of cell nuclei and over 95% reduction in DNA content to less than 50 ng/mg dry weight compared to native nerves, with no DNA fragments >200 bp in length, which meets the criteria for acellular tissues proposed by Crapo et al.^44^

The presence of well‐distributed essential ECM in ANAs provides the optimal substrate for Schwann cell migration and subsequent axon regrowth.^45^ Therefore, the preservation of ECM molecular composition is a critical requirement for decellularization. Collagen, as a major structural protein, constitutes about 49% of the total proteins of peripheral nerves. The ECM derived from nerves is predominantly composed of Collagen I that is involved in the fibril formation.^46^, ^47^ In the present study, although the histological analysis revealed that the morphological structure of M‐ANA prepared by the modified method has changed dramatically, these new ANAs maintained a comparable content of total collagen to that of the conventional ANAs. Also, the results of Western blot further confirmed that the levels of Collagen I were not significantly different between the two acellular groups, as well as Collagen IV. Remarkably, M‐ANA and S‐ANA were found to have higher contents of total collagen than native nerves. This can be explained by a significant reduction in tissue dry weight following decellularization, resulting in higher collagen ratios in acellular groups.^48^ Nevertheless, in terms of laminin and fibronectin which are prominent noncollagenous constituents of neural ECM, M‐ANA had significantly higher levels than S‐ANA. Furthermore, two decellularization methods also conveyed different conservation degrees of regenerative factors. More contents of VEGF, NGF, and BDNF were achieved in M‐ANA. Overall, the modified method effectively reduced the loss of ECM bioactive components and factors in contrast with the conventional method, which could be attributed to the application of mild detergents including the zwitterionic detergent CHAPS, as well as their lower working concentration and shorter incubation duration. The Sondell protocol is considered an aggressive decellularization approach, which tends to cause serious damage to ECM. In addition to the study of Hudson et al.,^20^ several recent studies have similarly introduced zwitterionic detergents, together with nonionic detergents or nucleases, to optimize nerve decellularization. These protocols showed reliable effects in reducing cellular components while maintaining ECM integrity.^49^, ^50^

GAGs are important ECM components in the peripheral nervous system that are implicated in the regulation of axon growth and guidance.^51^ The sulfated GAGs are covalently bound to core proteins of chondroitin sulfate proteoglycans (CSPGs) in their natural state.^52^ Due to their large size and negative charge, these sulfated GAGs can bind to growth‐promoting ECM molecules and suppress their function, which imparts axonal inhibitory activity to CSPGs.^53^ In the present study, the biochemical quantification revealed significant decreases of sulfated GAGs in M‐ANA and S‐ANA compared to native nerves. However, there were more sulfated GAGs found in M‐ANA than in S‐ANA, suggesting that the sulfated GAGs removal efficiency of the modified decellularization method is inferior to the conventional method, probably due to the application of SDC in the Sondell protocol. SDC, as a potent detergent, could lead to greater disruption of matrix components, especially GAGs.^54^ In the study of Philips et al., it can be observed that the decellularization method they devised incorporating Triton X‐100, DNase, and RNase but without SDC retained more sulfated GAGs than the Sondell method as well.^54^ Notably, although previous studies have demonstrated that the reduction of sulfated GAGs in ANAs by enzymatic treatment facilitated the repair of damaged nerves, excess degradation of sulfated GAGs has been noted to cause uncontrolled axonal sprouting outside nerve fascicles, resulting in inappropriate reinnervation.^55^, ^56^, ^57^ Moderate removal of sulfated GAGs may be a more feasible strategy to optimize ANAs rather than complete GAGs elimination.

The compact ultrastructure of ANAs derived from natural nerves may be optimal for supporting and protecting fully grown nerve fibers, but is theoretically less ideal for PNI repair.^25^ We have previously demonstrated that unidirectional freeze‐drying can induce the formation of continuous longitudinally aligned channels with pore sizes of 15–20 μm in ANAs.^30^ Therefore, this study integrated unidirectional freeze‐drying into our newly devised decellularization method. It was found that the microchannels in M‐ANA exhibited remarkably larger pore size than basal lamina tubes retained in S‐ANA, mainly ranging from 20 to 35 μm. The difference of pore sizes between our studies may be because the unidirectional freeze‐drying in this study was carried out subsequent to the detergent treatments, unlike the earlier study. Likewise, Sridharan et al. added unidirectional freeze‐drying after complete decellularization process, also generating microchannels with larger pore size in allografts (20–60 μm).^58^ The presence of large microchannels contributes to cell migration and axon growth during nerve repair. In particular, pore sizes of 20–35 μm in microchannels are considered an ideal compromise between axon extension and misdirection.^59^, ^60^ Additionally, due to the improvement of internal ultrastructure, M‐ANA achieved a significant increase in porosity, and exhibited greater swelling capacity compared to the conventional ANAs. This provides favorable conditions for the supply of nutrients and oxygen as well as metabolite clearance after transplantation and is important for the migration and survival of host cells.^61^ High porosity also facilitates the penetration and diffusion of reagents during decellularization, thus ensuring their functional performance and efficient elution.^62^

ANAs should provide sufficient mechanical strength for fixation and resistance to external forces. It should be noted that the mechanical environment can influence the behavior and functionality of neural cells within ANAs. It is necessary to match the approximate mechanical properties of target tissues.^63^, ^64^ The biomechanical analysis revealed that Young's modulus and stress at fracture of M‐ANA appeared to be elevated compared to native nerves, but not in a significant manner. However, these parameters in S‐ANA exhibited significantly higher levels than native nerves, suggesting an obvious difference in the effects of these two decellularization methods on the mechanical properties of allografts. Collagen fibers play a vital role in nerve elasticity.^65^ After decellularization, collagen fiber network would lose their intrinsic wave‐like pattern, resulting in the relaxation of collagen fibers.^66^, ^67^ Moreover, the removal of cells could enhance the mobility of collagen fibers, which allows them to reorient toward the direction of applied strain.^68^ These lead to changes in the mechanical features of ANAs. Previous studies also reported increased stiffness and ultimate stress in the allografts produced by the Sondell method.^69^ This may also be closely related to the disruption of ECM due to long‐time repeated treatments of SDC, especially the massive loss of GAGs.^54^ In contrast, the modified decellularization method was able to better maintain ECM and generate the multichannel ultrastructure, which endows the prepared allografts with mechanical performance closer to native nerves. Notably, the ultrastructure remodeling did not significantly alter the ability of ANAs to withstand suturing. The suture retention strength of M‐ANA was higher than the generally accepted adequate suture strength for implantation (2 N),^70^ with no significant difference to S‐ANA and native nerves.

Nerve regeneration takes place through the extension of Schwann cells rather than axon growth.^23^ Schwann cells guide and support the newborn axons, and exert key regulatory effects in the recovery process.^71^ The competence to support the viability and biological behavior of Schwann cells is essential for desirable nerve grafts. In the CCK8 assay, M‐ANA was demonstrated to be biocompatible, suggesting that almost no cytotoxic reagent residuals were present in the grafts. The DNA release assay further validated this result. Moreover, this novel nerve graft can support the growth and proliferation of Schwann cells as well as the conventional ANAs. More importantly, in contrast to S‐ANA, a large number of cells can be found to infiltrate into M‐ANA and migrate along the microchannels, which is largely attributed to the optimized multichannel ultrastructure introduced by the modified decellularization method. This finding is in agreement with other studies in which higher cell penetration was obtained in nerve grafts containing large microchannels.^60^, ^72^ In our earlier study, it was only at a later time point that Schwann cells were observed to start infiltrating into ANAs along the microchannels, probably because the improved pore size was mostly still less than 20 μm and thus difficult to permit effective cell infiltration.^30^ The diameters of Büngner's bands are mainly defined by axon diameter, which ranged from 2–5 μm to 15–20 μm.^73^ Previous studies have indicated that the microchannels with pore size above 20 μm are more conducive to the migration of Schwann cells,^25^, ^58^ and this study further confirms this view. In addition, although the RT‐PCR results did not show significant differences in the regulation of MPZ and NGFR p75 expression between the two acellular groups, a further increasing trend could be found in M‐ANA, likely associated with the greater preservation of ECM bioactive components and factors, which brings more possibilities for the full play of repair function of Schwann cells.

To evaluate the neurorestorative effects of the multichannel ANAs prepared by the modified decellularization method, we used these grafts to repair sciatic nerve defects in rats and harvested them at 6 and 12 weeks after surgery. It can be found that all the three groups had successfully bridged the nerve gaps. In M‐ANA, as with S‐ANA and ANG, the epineuriums at the junction of the grafts and the nerve stumps were smooth and continuous, without constriction or enlargement. During the extraction process, no graft disconnection occurred. These indicated that the connections formed by the novel ANAs and nerves can meet the fundamental mechanical need of transplantation. The in vivo experiments revealed that, at 6 weeks of grafting, M‐ANA could guide more nerve fibers into the allografts compared to S‐ANA. M‐ANA also had higher levels of myelination at both the central grafts and distal stumps. In particular, the nerve fibers guided by M‐ANA into distal segments exhibited greater myelin thickness and better G‐ratio. Myelin sheaths act as an electrical insulator to support the efficient conduction of neural signals and their thickness determines the conduction velocity of electrochemical information.^74^ Thicker myelin sheaths give rise to faster conduction. In addition, given that there is a trade‐off between myelin thickness and axon diameter due to spatial constraints, the parameter G‐ratio, reflecting their relationship, is also one of the reliable predictors to evaluate axon remyelination.^75^, ^76^ G‐ratio is informative of the underlying myelin ultrastructure, and could indicate the relative efficiency and maximal conduction velocity of axons.^77^ The optimal G‐ratio is thought to be around 0.6 in the peripheral nervous system.^78^ Myelinated axons with G‐ratio close to the ideal value may achieve higher conduction velocity and lower energy consumption, which contributes to the recovery of nerve function after injury.^79^ Indeed, MCV is demonstrated to be related to the myelination rate and regenerative axon level.^80^ The electrophysiological results revealed that M‐ANA did show a significant increase in MCV, closely resembling ANG, which is consistent with the histomorphological results. Notably, the ANAs produced using the Hudson method that better preserves ECM components and architecture were shown to have no significant advantage over S‐ANA in the early stage of nerve repair.^23^ This discrepancy may be ascribed to the presence of the optimized internal ultrastructure in M‐ANA. It is notable that acellular allografts need to take about 20 days before the first signs of vascularization in the midgraft appear. Prior to this, nutrient supply relies on fluid permeation from nerve stumps.^80^ The high porosity of M‐AMA could offer advantageous conditions for this process. Not only that, the microchannels with large pore size contribute to the early penetration of Schwann cells and rapid extension of axons. At 12 weeks of grafting, more myelinated axons were observed to extend across the allografts of M‐ANA and re‐enter the distal nerve segments than S‐ANA. More than that, the regenerative axons growing into the distal stumps exhibited better myelination quality in M‐ANA. Their G‐ratio is much closer to the ideal value of 0.6 with a significant increase in the thickness of myelin sheaths. The superior electrophysiological performances mean the efficient establishment of target organ reinnervation in M‐ANA, which leads to better restoration of muscle and motor function. Taken together, these results suggest that the novel ANAs possess a better capacity to facilitate nerve regeneration compared to the conventional ANAs, and exhibit a great potential for future clinical applications.

However, we also identified that there still exist gaps in regenerative performance between the optimized ANAs and autologous nerves. It requires a long way to transfer from bench to bedside. The novel ANAs can be recellularized to achieve further functionalization. The integration of neurotrophic factors may also be a feasible solution for improvement. In this study, unidirectional freeze‐drying was employed to impart axially aligned channels to ANAs. This multichannel structure could be further manipulated by modifying the freezing process, such as altering cold source and regulating temperature gradient, which may yield different impacts on nerve repair. More studies are required to determine the optimal ultrastructure for a growth‐permissive microenvironment. Moreover, the variations in nerve dimension and structure between different species are nonnegligible factors, which can greatly affect the successfulness of the decellularization process. This newly developed decellularization protocol should still be adapted and optimized to meet the needs of preparing human ANAs.

## 5 CONCLUSIONS

In conclusion, we developed a novel ANA with optimized multichannel ultrastructure using a modified decellularization method described in this study. These unique ANAs had significant advantages in the retention of main ECM bioactive molecules and regenerative factors, while eliminating over 95% of cellular components. The presence of the microchannels with larger pore size allowed them to obtain higher porosity and swelling performance. Their mechanical properties were more similar to those of native nerves. In addition, the optimized allografts exhibited an increased ability to support the proliferation and migration of Schwann cells in vitro. The in vivo assessment further demonstrated that compared to the conventional ANAs, they were more potent in promoting axon regrowth and myelination, thereby achieving better restoration of muscle and function. This study offers new insight into the improvement of ANAs, which brings greater likelihood for effectively treating PNI.

## AUTHOR CONTRIBUTIONS

**Tianhao Yu:** Conceptualization (equal); data curation (equal); formal analysis (equal); funding acquisition (lead); investigation (equal); methodology (equal); validation (equal); writing – original draft (equal); writing – review and editing (equal). **Qiang Ao:** Conceptualization (equal); data curation (equal); formal analysis (equal); funding acquisition (supporting); methodology (equal); writing – review and editing (equal). **Tianrang Ao:** Formal analysis (equal); methodology (equal); software (equal); validation (equal); writing – original draft (equal). **Muhammad Arslan Ahmad:** Conceptualization (equal); data curation (equal); methodology (equal); resources (equal); supervision (equal); validation (equal); writing – review and editing (equal). **Aijun Wang:** Formal analysis (equal); methodology (equal); resources (equal); writing – review and editing (equal). **Yingxi Xu:** Funding acquisition (equal); investigation (equal); methodology (equal); validation (equal); writing – original draft (equal); writing – review and editing (equal). **Zhongti Zhang:** Conceptualization (lead); data curation (lead); investigation (equal); project administration (supporting); resources (lead); software (lead); supervision (supporting); validation (supporting); writing – original draft (lead); writing – review and editing (supporting). **Qing Zhou:** Conceptualization (lead); funding acquisition (lead); methodology (lead); project administration (lead); resources (supporting); supervision (supporting); validation (supporting); writing – review and editing (supporting).

## CONFLICT OF INTEREST

All authors declare that there are no conflicts of interest related to this work.

### PEER REVIEW

The peer review history for this article is available at https://publons.com/publon/10.1002/btm2.10435.

## Supporting information


**APPENDIX S1** Supporting Information

## Data Availability

The data that support the findings of this study are available from the corresponding author upon reasonable request.
