# Supplementary material for: Preparation and assessment of an optimized multichannel acellular nerve allograft for peripheral nerve regeneration
Source: Bioeng Transl Med. 2022 Nov 1;8(4):e10435. doi: 10.1002/btm2.10435 (PMC10354778; doi:10.1002/btm2.10435)
Supplement: Supplementary file 1 — APPENDIX S1 Supporting Information [file BTM2-8-e10435-s001.docx]

**Supporting information for**

**Preparation and assessment of an optimized multichannel acellular nerve allograft for peripheral nerve regeneration**

Tianhao Yu, Qiang Ao, Tianrang Ao, Muhammad Arslan Ahmad, Aijun Wang, Yingxi Xu*, Zhongti Zhang*, Qing Zhou*

**The file includes:**

**SUPPLEMENTARY METHODS**

**SUPPLEMENTARY FIGURES**

**FIGURE S1**. The schematic diagram of the unidirectional freeze-drying process.

**SUPPLEMENTARY TABLES**

**TABLE S1**. Primer sequences used in this study

**TABLE S2**. The ratios of NF200-positive area at 6 and 12 weeks

**SUPPLEMENTARY METHODS**

**1. Isolation and culture of Schwann cells**

Schwann cells were harvested as described previously with minor modifications^1^ Briefly, sciatic nerves were excised from neonatal Sprague Dawley rats, and the epineurium was removed under a microscope. Then the sciatic nerves were cut into small pieces and incubated with collagenase (1%, Sigma-Aldrich, USA) and trypsin (0.125%, Sigma-Aldrich, USA) at 37℃ for 30 min. The mixture was triturated, centrifuged, and resuspended in Dulbecco’s modified Eagle’s medium/Ham’s F-12 50/50 Mix (DMEM/F12, Gibco, USA) containing 10% fetal bovine serum (FBS, Gibco, USA) and 1% penicillin/streptomycin (Gibco, USA). The cell suspension was placed into poly-L-lysine pre-coated dishes and incubated in a 37°C humidified incubator with 5% CO_2_. After 24 h, Schwann cells were treated with the medium containing 10 μM cytosine arabinoside (Sigma-Aldrich, USA) twice for 48 h to remove rapidly proliferating fibroblasts. After purified Schwann cells reached confluence, they were passaged, split, and re-plated. The medium was changed every 2 days. Schwann cells were passaged no more than 4 times.

**2. Identification of Schwann cells**

The extracted primary Schwann cells were identified by immunofluorescence staining for S-100, the Schwann cell-specific marker. Immunofluorescence staining was carried out according to the standard protocol^2^. Briefly, the cells were fixed with 4% paraformaldehyde for 10 min, permeabilized with 0.1% Triton X-100 for 10 min, and then blocked with normal goat serum for 30 min. Next, the cells were treated with anti-S-100 antibody (1:200, Abcam, USA) at 4°C overnight, followed by incubation with fluorescent-conjugated secondary antibodies (1:1000, Abcam, USA) for 1 h at 37°C. The nuclei were stained with DAPI (Sigma-Aldrich, USA). The images of labeled cells were captured with a laser scanning confocal microscopy (FV1200, Olympus, Japan).

**REFERENCES**

1 Ao Q, Fung CK, Tsui AY, et al. The regeneration of transected sciatic nerves of adult rats using chitosan nerve conduits seeded with bone marrow stromal cell-derived Schwann cells. *Biomaterials*. 2011;32(3):787-96.

2 Zhou LN, Wang JC, Zilundu PLM, et al. A comparison of the use of adipose-derived and bone marrow-derived stem cells for peripheral nerve regeneration in vitro and in vivo. *Stem cell research & therapy*. 2020;11(1):153.

**SUPPLEMENTARY FIGURES**


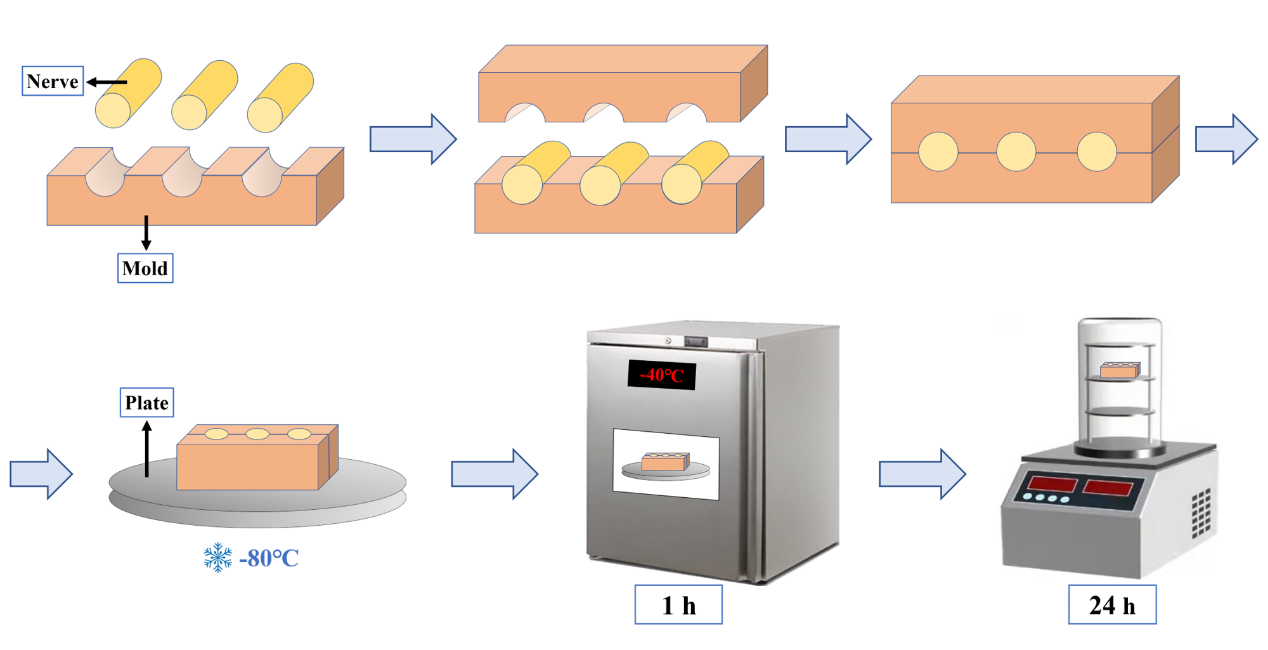


**FIGURE S1.** The schematic diagram of the unidirectional freeze-drying process. The procedure is to first put nerve segments into the prefabricated grooves in one side of the mold, and then close the mold. Subsequently, the mold containing nerve segments was placed on the precooled stainless steel plate, and rapidly transferred to a freezer (-40°C). After a 1 h hold period, the mold was moved to a freeze dryer and lyophilized for 24 h to remove ice crystals and generate microchannels.

**SUPPLEMENTARY TABLES**

**TABLE S1.** Primer sequences used in this study

| Target genes | Forward primer (5′-3′) | Reverse primer (5′-3′) |
| --- | --- | --- |
| GAPDH | ATGGTGAAGGTCGGTGTGAACG | TTACTCCTTGGAGGCCATGTAG |
| MPZ | GCTCTTCTCTTCTTTGGTGCTGTCC | GGCGTCTGCCGCCCGCGCTTCG |
| NGFR p75 | ATGAGGAGGGCAGGTGCTG | TCACACTGGGGACGTGGC |

**TABLE S2.** The ratios of NF200-positive area at 6 and 12 weeks

Data are expressed as the mean ± SD (n = 8). **p* < 0.05 compared to ANG, ^#^*p* < 0.05 compared to S-ANA.

|  |  | 6 weeks (%) | |  | 12 weeks (%) | |
| --- | --- | --- | --- | --- | --- | --- |
| Groups |  | Central | Distal |  | Central | Distal |
| ANG |  | 7.26±0.84 | 6.14±0.80 |  | 11.41±1.18 | 9.64±0.81 |
| S-ANA |  | 4.75±0.60 * | 3.90±0.90 * |  | 7.45±1.00 * | 5.50±0.94 * |
| M-ANA |  | 6.12±0.66 * ^#^ | 4.82±1.00 * |  | 9.48±1.29 * ^#^ | 7.25±0.89 * ^#^ |
